# Olfactory impairment in Wilson’s disease

**DOI:** 10.1002/brb3.2022

**Published:** 2021-01-07

**Authors:** Lei Chen, Xin Wang, Richard L. Doty, Shanshan Cao, Junxiu Yang, Feng Sun, Xiaoyan Yan

**Affiliations:** ^1^ Department of Neurology Tianjin Huan Hu Hospital Tianjin Key Laboratory of Cerebrovascular and Neurodegenerative Diseases Tianjin China; ^2^ Smell and Taste Center Perelman School of Medicine University of Pennsylvania Philadelphia PA USA; ^3^ Department of Gerontology The No. 2 Hospital of Baoding Baoding China; ^4^ Department of Neurology Hebei Petro China Central Hospital Langfang China; ^5^ Department of Neurology Tianjin Medical University General Hospital Tianjin China; ^6^ Peking University Clinical Research Institute Peking University First Hospital Beijing China

**Keywords:** diagnosis, olfactory function, Wilson’s disease

## Abstract

**Introduction:**

Olfactory dysfunction is a common and early sign of many neurodegenerative disorders, but little is known about olfactory dysfunction in Wilson’s disease (WD). We aimed to evaluate olfactory function in patients with WD and identify selective WD screening odors.

**Methods:**

We measured olfactory identification ability in 25 patients with WD and 25 healthy controls using the University of Pennsylvania Smell Identification Test (UPSIT). Patients with WD were evaluated using the Global Assessment Scale for WD (GAS). Cognitive function was measured using the Mini–Mental State Examination.

**Results:**

Patients with WD were worse at identifying smells in the simplified Chinese version of the UPSIT compared with healthy controls (*t* = 2.198, *p* = .033), but there was no difference in olfactory dysfunction severity between the groups (*V* = 136, *p* = .094). UPSIT scores negatively correlated with the GAS neurological scores in patients with WD (*r* = −0.571, *p* = .003). Using logistic regression with least absolute shrinkage and selection operator analysis, two models were screened. Receiver‐operating characteristic (ROC) curve analysis revealed that, to discriminate WD patients from healthy controls, the area under the ROC curve (AUC) for a combination of seven odors (motor oil, onion, licorice, strawberry, tire, jasmine, and natural gas) was 0.926, while the AUC for three odors (onion, licorice, and jasmine) was 0.852.

**Conclusions:**

Patients with WD may have stable, selective olfactory impairments. This selective pattern may be a useful tool for disease diagnosis and prediction.

## Graphical abstract

Olfactory function and selective odor identification impairment has been explored in patients with WD. We found that the patients with WD were worse at identifying smells compared with healthy controls, but the hyposmia severity was milder than that found in PD or AD patients. A selective pattern of olfactory dysfunction was also detected in WD, and both the seven odor model and the three odor model showed good disease discrimination accuracy.

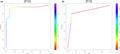

## 1 INTRODUCTION

Wilson’s disease (WD) is an autosomal recessive disorder of copper (Cu) metabolism, caused by mutations in the ATP7B gene, which encodes the Cu transporting ATPase expressed mainly in hepatocytes. ATP7B is a multifunctional membrane‐spanning protein which contributes to the production of ceruloplasmin and expedites biliary excretion of copper when intracellular copper concentrations are elevated (Bandmann et al., 2015). The malfunction of the ATP7B leads to copper accumulating in the hepatocytes, leaking into circulation and ultimately affecting other organs, notably the central nervous system. As a result, WD can present with a variety of clinical patterns with the most prominent symptoms of cirrhosis, neurologic dysfunction and psychiatric features. Parkinsonism is a common manifestation in patients with WD (Członkowska et al., 2017).

Olfactory dysfunction is a common sensory symptom in neurodegenerative disorders such as Parkinson's disease (PD), Alzheimer's disease (AD), and hereditary ataxia (Boesveldt et al., 2008; Doty, 2017; Fernandez‐Ruiz et al., 2003; Tabert et al., 2005). Olfaction is maintained by the olfactory nerve, along with the olfactory bulb and tract, orbitofrontal region, insula, orbitofrontal cortex, amygdala, and cerebellum, which is organized as an olfactory network. The striatum and the thalamus are also involved in odor processing (Hawkes & Doty, 2018). The neuropathological hallmark of WD is neuronal loss and atrophy in the lenticular nucleus, as well as in the midbrain, thalamus, and other parts of the basal ganglia (Meenakshi‐Sundaram et al., 2008). This involvement of the olfactory network may lead to olfactory dysfunction in WD. Another potential mechanism on olfactory function among WD is that Cu can impair the structural, regulatory, and catalytic functions of different enzymes, proteins, receptors, and transporters (Mezzaroba et al., 2019). It had been demonstrated that copper played an essential role in detection of methanethiol by olfactory receptor MOR244‐3 in mice (Block et al., 2017). Taken together, WD may develop olfaction dysfunction in common with other neurodegenerative disorders. Hyposmia was first studied in small populations of patients with WD, and patients with neurological symptoms showed significant olfactory dysfunction compared with hepatic‐type patients. Moreover, individuals with more severe neurologically affected also presented with a more pronounced olfactory deficit (Mueller et al., 2006). However, olfactory impairment in WD has not yet been intensively studied.

In previous studies, selective deficits of odor identification have been identified in neurodegenerative disorders. For example, Kelvin et al. reported that three specific odors (banana, licorice, and dill pickle) were able to distinguish PD subjects from controls with high specificity (Bohnen et al., 2007). Another study reported that two odors from the University of Pennsylvania Smell Identification Test (UPSIT), pizza and wintergreen, were able to distinguish PD patients from controls (Hawkes & Shephard, 1993). Such selective odor identification impairments have also been reported in AD; using the UPSIT, Tabert et al. identified 10 odors that were selectively associated with AD risk (Tabert et al., 2005). However, although patients with WD may perform more poorly than healthy controls, a detailed impairment pattern for odor identification has never been investigated in this population in the past. The characterization of this pattern may allow for the development of more specific diagnostic olfactory tests for WD, compared with the current functional assessment of global odor identification. Therefore, the aim of the present study was to investigate olfactory function and assess the pattern of odor identification impairment in patients with WD.

## 2 MATERIAL AND METHODS

### 2.1 Participants

Twenty‐five patients with WD were recruited from the outpatient clinic of the Department of Neurology of Tianjin Union Medical Center and Tianjin Huan Hu Hospital (Tianjin, China). The diagnosis of WD was based on the combination of the following: the presence of Kayser–Fleischer rings detected by slit lamp examination, increased 24‐hr urinary copper excretion (>100 mcg/24 hr), decreased serum ceruloplasmin level (<20 mg/dl), and hepatic changes identified by biochemical and ultrasound evidence. All patients had symptomatic neurological involvement and had been on a stable dose of D‐penicillamine treatment for at least 6 months without neurological worsening. Patients were evaluated by an experienced neurologist. The exclusion criteria were as follows: (a) patients who had undergone a liver transplant prior to inclusion; (b) patients with concomitant systemic diseases or who were taking drugs that can affect olfaction; (c) patients with Mini–Mental State Examination (MMSE) scores lower than 26; and (d) patients with head trauma, psychiatric disorders (psychosis), other neurological diseases, or congenital olfactory disorders. Furthermore, 25 healthy controls, recruited from a similar area and matched by sex and age (range ±5 years), were enrolled from the Medical Examination Clinic of Tianjin Union Medical Center. All participants received brief interviews and physical examinations to rule out any conditions that can cause olfactory dysfunction, including nasal septum deviation, nasal polyposis, allergic rhinitis, acute or chronic rhinosinusitis, previous nasal or paranasal surgery, recent upper airway infections, and smoking.

### 2.2 Evaluation protocol

The MMSE was used to assess global cognitive abilities. Odor identification was assessed using the simplified Chinese version of the UPSIT (Doty et al., 1984; Jiang et al., 2010). The UPSIT is a widely used 40‐item “scratch and sniff” tool for identifying individuals with olfactory dysfunction from healthy controls. After scratching the scent area, the participant selects the smell from four options in a forced‐choice paradigm. A higher score (out of 40 points) indicates better odor identification. It has been developed into multiple cultures and languages, including the simplified Chinese version. Following the normative data presented in the UPSIT manual, which include adjustments for age and sex, scores ≥31–33 in males and 31–34 in females were considered to reflect normosmia, while scores ≤18 reflected anosmia. Hyposmia was further subdivided into mild, moderate, and severe forms according to the UPSIT manual. The severity and profile of WD were quantified using the Global Assessment Scale for WD (GAS; Aggarwal et al., 2009). The GAS is composed of two parts: Tier 1 and Tier 2. Tier 1 reflects global disability, whereas Tier 2 reflects neurological impairment. Both Tier 1 and Tier 2 were evaluated by an experienced neurologist.

### 2.3 Statistical analysis

Statistical analyses of demographic, clinical, and olfactory data were performed using IBM SPSS Statistics 22.0.0 (2013; IBM Corp) and Statistical Analysis System (SAS; version 9.3, SAS Institute Inc.) software. The results are presented as means with standard deviations or as percentages. Group differences in demographic and clinical variables between healthy controls and WD patients were analyzed using independent *t*‐tests for quantitative data or with chi‐squared tests for categorical data. The Mann–Whitney *U* test was used when data did not follow a normal distribution. Matched ordinal statistical tests were performed to compare the severity of olfactory dysfunction (anosmia; severe, moderate, or mild hyposmia; or normosmia) between healthy controls and patients with WD. The 40 odor variables from the UPSIT were submitted to logistic regression with least absolute shrinkage and selection operator (LASSO) to screen and cross‐validate possible disease‐specific odors for WD. The selected values were then applied to receiver‐operating characteristic (ROC) curves to evaluate the accuracy of a combination of selected odors for the discrimination of WD from healthy controls. To study the relationship between clinical characteristics and olfactory identification function, the Pearson's correlation coefficient test was used. Statistical significance was set at *p* < .05.

### 2.4 Ethical statement

The study was approved by the Ethics Committee of Tianjin Huan Hu Hospital according to the Declaration of Helsinki. Verbal consent was obtained from all participants. The need for written consent was waived because the research presented no risk of harm to the subjects and did not involve identifiable private information.

## 3 RESULTS

### 3.1 Demographic and clinical characteristics

There were no significant differences between the two groups in age (*t* = 0.123, *p* = .903) or years of education (*t* = 0.238, *p* = .813). Patients with WD had lower MMSE scores compared with healthy controls (*z* = −3.053, *p* = .002), although the median score of both groups was 29.00. The mean GAS Tier 1 score was 3.72 ± 1.137 and the mean GAS Tier 2 score was 10.40 ± 4.113 (Table 1).

**TABLE 1:** Demographic and clinical characteristics in Wilson’s disease (WD) patients and healthy controls

	HC (*n* = 25)	WD (*n *= 25)	*t*/*z*	*p*
Ages (years)	24.20 ± 8.226	23.92 ± 7.868	0.123	.903
Sex (male)	15	15	—	—
Age of onset (years)	—	13.72 ± 5.594	—	—
Disease duration (years)	—	10.16 ± 8.245	—	—
Years of education	9.76 ± 2.454	9.60 ± 2.291	0.238	.813
GAS Tier1	—	3.72 ± 1.137	—	—
GAS Tier2	—	10.40 ± 4.113	—	—
MMSE	29.00(29.00,30.00)	29.00(26.50,29.00)	−3.053	.002**
UPSIT	31.60 ± 3.33	28.56 ± 6.06	2.198	.033*

GAS, Global Assessment Scale for WD; HC, healthy controls; MMSE, Mini–Mental State Examination; WD, Wilson’s disease.

Variables are expressed as the mean (standard deviation), median (interquartile range), or *n* (%). *p < .05; **p < .01; ***p < .001.

### 3.2 Objective olfactory performance

**FIGURE 1:**
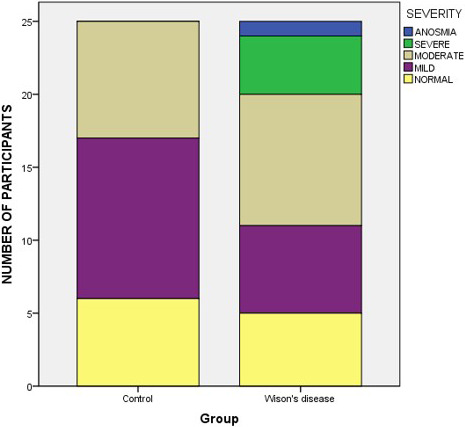
Severity of odor identification dysfunction in patients with Wilson’s disease (WD) and healthy controls (*V* = 136, *p* = .094)

**FIGURE 2:**
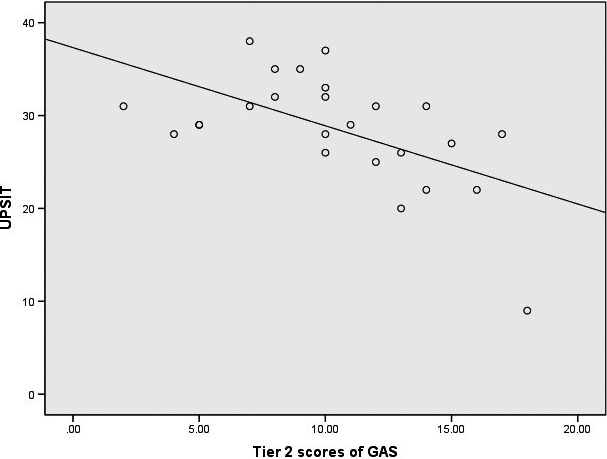
Negative correlation between the severity of neurological impairment and olfactory function in Wilson’s disease (WD). GAS, Global Assessment Scale for WD; UPSIT, University of Pennsylvania Smell Identification Test

Patients with WD were significantly worse at identifying smells on the UPSIT compared with healthy controls (28.56 ± 6.06 vs. 31.60 ± 3.33, *t* = 2.198, *p* = .033; Table 1). Based on the age‐ and sex‐based normative values provided in the UPSIT manual, five WD patients had normal odor identification, while six patients had mild hyposmia, nine had moderate hyposmia, four had severe hyposmia, and one was anosmic. Six healthy control subjects had normal odor identification, while there were eleven mild and eight moderate cases of hyposmia; no severe hyposmia or anosmia was identified in the healthy control group. There was no significant difference in the severity of odor identification impairment between the two groups using a Wilcoxon signed rank test after matching for age and sex (*V* = 136, *p* = .094; Figure 1). In WD patients, UPSIT scores were significantly negatively correlated with the Tier 2 (neurological assessment section) GAS scores (*r* = −0.571, *p* = .003; Figure 2), but were not significantly correlated with age, age of onset, MMSE scores, years of education, or years of disease duration (Table 2).

**TABLE 2:** Correlation between olfactory identification (University of Pennsylvania Smell Identification Test [UPSIT] scores) and clinical characteristics in Wilson’s disease (WD) patients

Clinical characteristics	*r*	*p*
Age	−.132	.362
Age of onset (years)	.196	.347
Disease duration (years)	−.364	.073
MMSE	.141	.501
Years of education	.031	.829
GAS Tier 2	−.571	.003**

GAS, Global Assessment Scale for WD; MMSE, Mini–Mental State Examination.

* *p* < .05; **p < .01.

**FIGURE 3:**
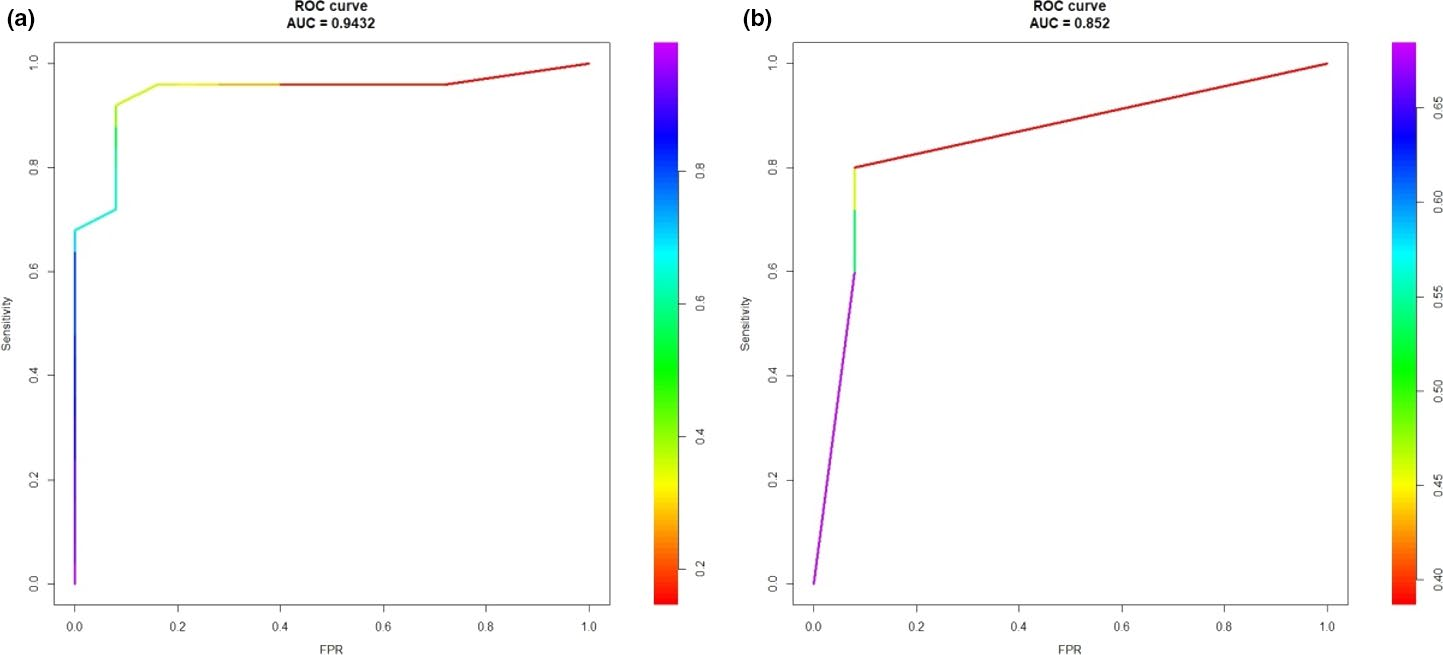
Receiver‐operating characteristic (ROC) curves for the discrimination of WD using a combination of either seven or three items from the UPSIT. (a) The seven odor model (motor oil, onion, licorice, strawberry, tire, jasmine, and natural gas), AUC = 0.943. (b) The three odor model (onion, licorice, and jasmine), AUC = 0.852

In a logistic regression with LASSO analysis, two models were chosen based on cross‐validation. The first model contained seven odor variables, based on the variables that had the best average performance during cross‐validation, and the second model contained the three odor variables that had the least number of variables for efficiency in future odor testing while retaining a high area under the ROC curve (AUC) score (>0.8). The model that included seven odor variables (motor oil, onion, licorice, strawberry, tire, jasmine, and natural gas) showed discrimination accuracy for WD from healthy controls with an AUC of 0.943, whereas the model that included three odor variables (onion, licorice, and jasmine) showed discrimination accuracy with an AUC of 0.852 (Table 3; Figure 3).

**TABLE 3:** Odors selected by the logistic regression with LASSO

Item	Seven odor model		Three odor model	
	*z*	*p*	*z*	*p*
Motor oil	−1.781	.075	—	—
Onion (Correct/Incorrect)	−3.135	.002**	−3.934	<.001***
Licorice (Correct/Incorrect)	−0.005	.996	−0.007	.994
Strawberry	1.114	.265	—	—
Tire	−2.140	.032*	—	—
Jasmine	−0.005	.996	−0.007	.994
Natural gas	−1.796	.073	—	—

LASSO, least absolute shrinkage and selection operator.

* *p* < .05; **p < .01; ***p < .001.

## 4 DISCUSSION

In the current cohort of WD patients with a clear, predominant neurological manifestation, we found that smell identification function was significantly impaired compared with matched, healthy controls; there was a higher frequency of moderate or severe hyposmia and anosmia in the patients with WD, although this difference in the severity of olfactory dysfunction did not reach statistical significance between the two groups (*p* = .094). The severity of hyposmia was significantly correlated with neurological impairment in the WD patients, but was not correlated with age, age of onset, disease duration, years of education, or MMSE scores. We did not take D‐penicillamine treatment into consideration as an influencing factor because a previous study reported that penicillamine has no significant effect on olfactory function (Meenakshi‐Sundaram et al., 2008). The results from our study suggest that olfactory impairment may be a common and stable symptom in WD, as it is in PD (Boesveldt et al., 2008). However, the severity of odor identification impairment among WD patients was milder than that found in PD or AD patients, where the UPSIT scores are typically in the low 20s (vs. 28.56 ± 6.06 in our study; Barrios et al., 2007; Doty & Hawkes, 2019; Doty et al., 1991). A recent study has reported that WD patients consistently had olfactory dysfunction compared with controls, but the difference between groups was only slight (5.9 ± 0.2 vs. 6.3 ± 0.3), and sub‐item scores, including for odor threshold and odor identification, were similar (Degirmenci et al., 2019). These findings imply that hyposmia is a common symptom of WD, but that it is far less marked than in PD or AD.

We also revealed a selective pattern of olfactory dysfunction in WD, and both the seven odor model and the three odor model showed good disease discrimination accuracy (AUC = 0.943 and 0.852, respectively). Several previous studies have demonstrated the apparent selectivity of olfactory dysfunction in PD and AD (Bohnen et al., 2007; Hawkes & Shephard, 1993; Tabert et al., 2005), but no characteristic pattern of odor discrimination has previously been investigated in patients with WD. Our findings suggest that hyposmia in WD may present a specific feature, as has been previously observed in PD, and that a brief version of a disease‐specific odor screening test may contribute to diagnosis and lower the cost of olfactory evaluations in WD.

Odor identification performance is a complex task requiring the involvement of both olfactory and cognitive pathways (Schubert et al., 2013). Thus, cognitive impairment may lead to olfactory information processing deficits, even though the olfactory anatomical structures and circuits are intact and normal. In our study, the patients with WD had worse global cognitive scores than the healthy controls, but they all presented normal global cognitive function (MMSE scores ≥26) and there was no correlation between cognitive function and smell identification in the patients. Hyposmia in WD in this study may therefore mainly result from deficits in olfactory‐related neural pathways rather than from the abnormal cognitive processing of olfactory information, because the effects of cognitive factors on olfactory function were restricted.

The limitations of this study should be kept in mind when interpreting the results. First, our sample size was relatively small, and subjects were predominantly male. Women generally perform better than men in odor identification testing (Sorokowski et al., 2019), and this bias might have caused the average UPSIT scores in our study to be lower than those in the general population. Second, the UPSIT scores of the controls were lower than the scores reported in many other studies. The simplified Chinese version of the UPSIT has been developed from the Northern American version (UPSIT‐NA), which might have a cultural bias. In a previous UPSIT‐NA study carried out in 40 young, healthy Taiwanese subjects, 10 odorants were correctly identified by less than two‐thirds of the subjects (Jiang et al., 2002). In the present study, nine items (pizza, cherry, fruit punch, fish, strawberry, grass, popcorn, orange, and pine) had an error rate of more than 70% in the control group. Moreover, the standard we used to classify smell dysfunction was based on the UPSIT‐NA manual, because no cutoff values were available for the simplified Chinese version. Thus, cultural differences and our predominantly male sample may account for the lower UPSIT scores of the controls. However, considering the aim of our study, we were able use the control scores as a reference to explore olfactory function in patients with WD. Third, structural or functional investigations of the olfactory network were not performed using any imaging techniques. Olfactory processing involves multiple anatomical areas and functional connectivity, and these complex interrelations make it difficult to attribute the observed olfactory deficits to any specific structures. Thus, we hadn’t explored the underlying brain lesions accounting for olfactory impairment in this preliminary study.

## 5 CONCLUSIONS

In conclusion, WD patients may have a selective, stable olfactory dysfunction that is milder than that found in PD or AD. A WD‐specific odor screening test might be a useful tool for disease diagnosis, but further studies need to be conducted to confirm and elucidate our findings.

## CONFLICT OF INTEREST

Dr. Doty is a consultant to Acorda Therapeutics, Eisai Co, Ltd, Merck Pharmaceuticals, the Michael J. Fox Foundation for Parkinson’s Research, and Johnson & Johnson. He receives royalties from Cambridge University Press, Johns Hopkins University Press, and John Wiley & Sons, Inc. He is president of, and a major shareholder in, Sensonics International, a manufacturer and distributor of smell and taste tests, including the test used in this study.

## AUTHORS’ CONTRIBUTIONS

All authors had complete access to all study data and assume complete responsibility for the integrity of the data and accuracy of the data analysis. LC and XW recruited the patient, wrote the first draft of the manuscript, reviewed the literature and revised the draft. They, LC and XW, contributed to the work equally and should be regarded as co‐first authors. RD provided with UPSIT booklets and participated in the study design. SC, JY and FS completed the evaluations of the participants. XY conducted the statistical analysis. All authors have read and approved the final manuscript.

## CONSENT FOR PUBLICATION

Verbal consent was obtained from all participants.

### Peer Review

The peer review history for this article is available at https://publons.com/publon/10.1002/brb3.2022.

## Data Availability

Dataset available on reasonable request from the corresponding author.
